# Predictive value of neutrophil to apolipoprotein A1 ratio in patients with acute ischaemic stroke

**DOI:** 10.1093/braincomms/fcae091

**Published:** 2024-03-21

**Authors:** Chao Chen, Shengqi Li, Fangyue Sun, Yiqun Chen, Haojie Qiu, Jiaqi Huang, Yining Jin, Jiexi Huang, Jiahan Xu, Zerui Jiang, Kun Li, Yanchu Wang, Hai Lin

**Affiliations:** 1 Department of Nutriology, The Third Affiliated Hospital of Wenzhou Medical University, 325200 Wenzhou, China; 1 Department of Nutriology, The Third Affiliated Hospital of Wenzhou Medical University, 325200 Wenzhou, China; The First School of Medicine, School of Information and Engineering, Wenzhou Medical University, 325035 Wenzhou, China; 1 Department of Nutriology, The Third Affiliated Hospital of Wenzhou Medical University, 325200 Wenzhou, China; The First School of Medicine, School of Information and Engineering, Wenzhou Medical University, 325035 Wenzhou, China; 1 Department of Nutriology, The Third Affiliated Hospital of Wenzhou Medical University, 325200 Wenzhou, China; The First School of Medicine, School of Information and Engineering, Wenzhou Medical University, 325035 Wenzhou, China; 1 Department of Nutriology, The Third Affiliated Hospital of Wenzhou Medical University, 325200 Wenzhou, China; The Second School of Medicine, Wenzhou Medical University, 325035 Wenzhou, China; 1 Department of Nutriology, The Third Affiliated Hospital of Wenzhou Medical University, 325200 Wenzhou, China; The First School of Medicine, School of Information and Engineering, Wenzhou Medical University, 325035 Wenzhou, China; 1 Department of Nutriology, The Third Affiliated Hospital of Wenzhou Medical University, 325200 Wenzhou, China; The Second School of Medicine, Wenzhou Medical University, 325035 Wenzhou, China; 1 Department of Nutriology, The Third Affiliated Hospital of Wenzhou Medical University, 325200 Wenzhou, China; The First School of Medicine, School of Information and Engineering, Wenzhou Medical University, 325035 Wenzhou, China; 1 Department of Nutriology, The Third Affiliated Hospital of Wenzhou Medical University, 325200 Wenzhou, China; The First School of Medicine, School of Information and Engineering, Wenzhou Medical University, 325035 Wenzhou, China; 1 Department of Nutriology, The Third Affiliated Hospital of Wenzhou Medical University, 325200 Wenzhou, China; The First School of Medicine, School of Information and Engineering, Wenzhou Medical University, 325035 Wenzhou, China; 1 Department of Nutriology, The Third Affiliated Hospital of Wenzhou Medical University, 325200 Wenzhou, China; The First School of Medicine, School of Information and Engineering, Wenzhou Medical University, 325035 Wenzhou, China; 1 Department of Nutriology, The Third Affiliated Hospital of Wenzhou Medical University, 325200 Wenzhou, China; The First School of Medicine, School of Information and Engineering, Wenzhou Medical University, 325035 Wenzhou, China; Department of Endocrinology, The Third Affiliated Hospital of Wenzhou Medical University, 325200 Wenzhou, China

**Keywords:** stroke, acute ischaemic stroke, prognosis, neutrophil to apolipoprotein A1 ratio, after intravenous thrombolysis

## Abstract

The neutrophil to apolipoprotein A1 ratio has emerged as a possible prognostic biomarker in different medical conditions. Nonetheless, the predictive potential of neutrophil to apolipoprotein A1 ratio in determining the 3-month prognosis of acute ischaemic stroke patients who undergo intravenous thrombolysis has yet to be fully acknowledged. In this study, 196 acute ischaemic stroke patients with recombinant tissue plasminogen activator and 133 healthy controls were included. Meanwhile, we incorporated a total of 386 non-thrombolytic acute ischaemic stroke patients. The acute ischaemic stroke patients with recombinant tissue plasminogen activator were divided into four groups based on quartiles of neutrophil to apolipoprotein A1 ratio. The association between neutrophil to apolipoprotein A1 ratio and the 3-month prognosis was evaluated through univariate and multivariate regression analyses. Additionally, subgroup analyses were conducted to investigate the predictive value of neutrophil to apolipoprotein A1 ratio in different patient populations. Adverse outcomes were defined as a modified Rankin Scale score of 3–6. The study findings revealed a significant association between elevated neutrophil to apolipoprotein A1 ratio levels and poor prognosis in acute ischaemic stroke patients. In the highest quartile of neutrophil to apolipoprotein A1 ratio levels (Q4), after controlling for age, gender, admission National Institutes of Health Stroke Scale score, blood urea nitrogen and stroke subtypes, the odds ratio for adverse outcomes at 3 months was 13.314 (95% confidence interval: 2.878–61.596, *P* = 0.001). An elevated neutrophil to apolipoprotein A1 ratio value was found to be associated with a poor prognosis in acute ischaemic stroke patients, regardless of whether they received recombinant tissue plasminogen activator treatment or not. The new model, which incorporating neutrophil to apolipoprotein A1 ratio into the conventional model, demonstrated a statistically significant improvement in discriminatory power and risk reclassification for 3-month poor outcomes in acute ischaemic stroke patients treated with recombinant tissue plasminogen activator. The new model exhibited a categorical net reclassification index (*P* = 0.035) of 12.9% and an integrated discrimination improvement (*P* = 0.013) of 5.2%. Subgroup analyses indicated that the predictive value of neutrophil to apolipoprotein A1 ratio differed across stroke subtypes. Neutrophil to apolipoprotein A1 ratio is a potential biomarker for predicting the prognosis of acute ischaemic stroke patients. The clinical implications of our findings are significant, as early identification and intervention in high-risk patients can improve their outcomes. However, further studies are required to validate our results and elucidate the underlying mechanisms of the association between neutrophil to apolipoprotein A1 ratio and poor prognosis in acute ischaemic stroke patients.

## Teaser

Chen *et al*. investigated the predictive value of the neutrophil to apolipoprotein A1 ratio (NAR) for 3-month outcomes in acute ischaemic stroke with recombinant tissue plasminogen activator treatment. They suggested that NAR could potentially serve as a promising biomarker for predicting prognosis in stroke patients.

## Graphical Abstract

**Graphical Abstract:**
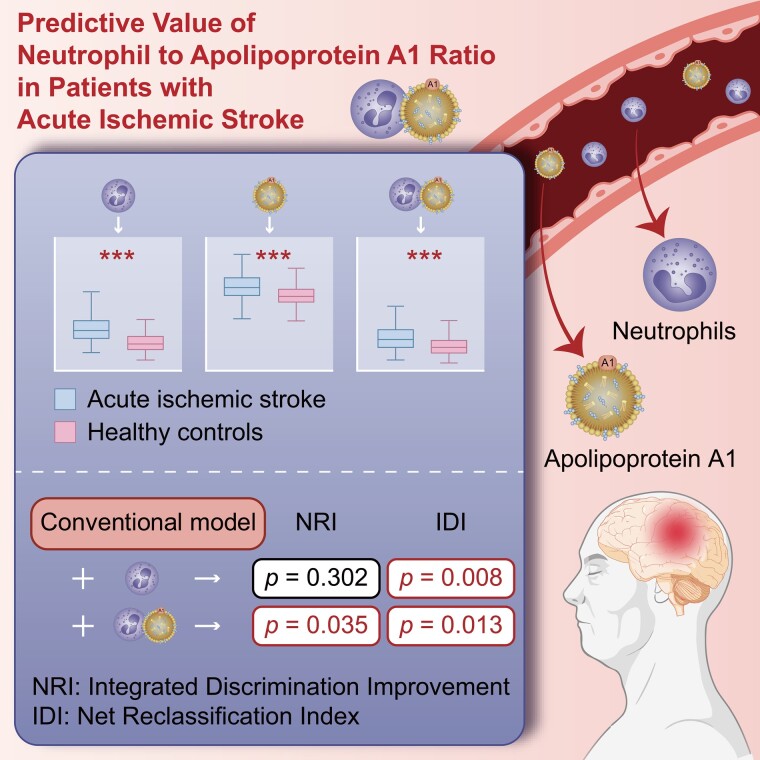

## Introduction

As one of the three major diseases that threaten human health, stroke is considered the second leading cause of death and the third leading cause of disability worldwide.^1^ Current treatment strategies for acute ischaemic stroke (AIS) mainly focus on recanalization techniques, including intravenous or intraarterial administration of recombinant tissue plasminogen activator (r-tPA) and mechanical endovascular therapies.^2^ However, these methods inevitably result in poor prognosis. Therefore, it is crucial to explore the key prognostic factors that can predict the prognosis earlier to prevent adverse outcomes.

Stroke results in brain injury through mechanisms that involve oxidative stress and a complex immune response.^3-6^ Within the first few hours after stroke onset, neutrophils are recruited to ischaemic sites and release chemical mediators that exacerbate tissue damage and poor neurologic improvement, potentially leading to adverse clinical outcomes.^7^ High density lipoprotein (HDL) is a complex lipoprotein composed of proteins and lipids with pleiotropic effects, including antioxidant, anti-apoptotic, anti-inflammatory, anti-thrombotic and anti-proteolytic properties.^8^ Apolipoprotein A1 (ApoA1), the major apolipoprotein component of HDL cholesterol (HDL-C), possesses both anti-inflammatory and antioxidant properties.^9^ ApoA1 is also widely used in clinical practice to estimate the risk of cerebrovascular disease, with a decrease in its level associated with an increased risk of such disease.^10^

Previous studies have shown that the neutrophil to apolipoprotein A1 ratio (NAR) is an independent indicator for predicting overall survival in patients with hepatocellular carcinoma and nasopharyngeal carcinoma.^11,12^ However, the correlation between NAR and cerebral infarction has not been studied yet. Therefore, in this retrospective observational study, our objective was to investigate whether NAR can predict and evaluate the 3-month prognosis of AIS patients treated with intravenous thrombolysis using r-tPA.

## Materials and methods

### Study population

**Figure 1:**
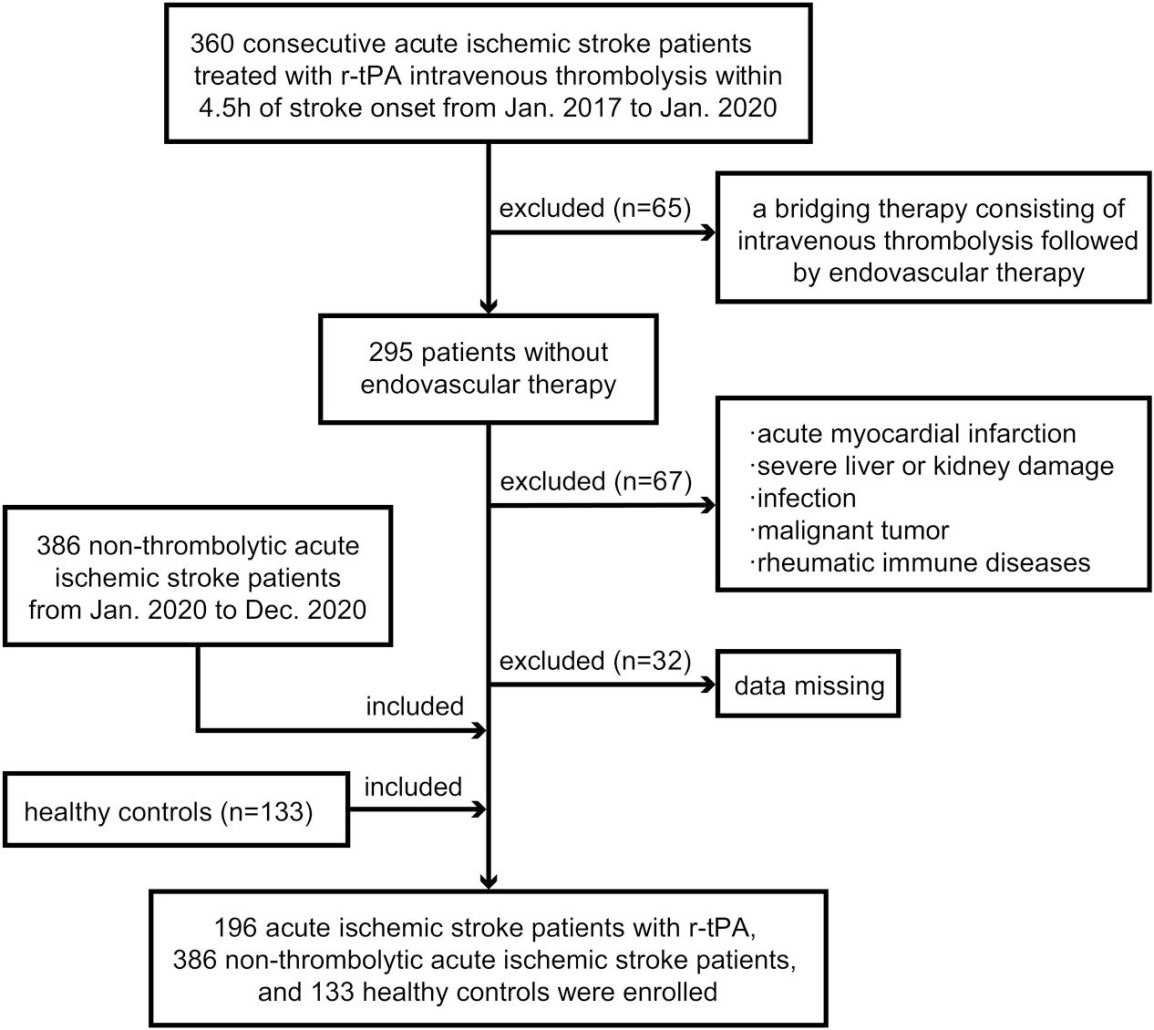
**Flow diagram showing the patient selection process.** r-tPA, recombinant tissue plasminogen activator.

The population included in this study is shown in Fig. 1 in the form of a flow chart. From January 2017 to January 2020, 360 patients diagnosed with AIS and treated with thrombolytic therapy using r-tPA were consecutively recruited from the Third Affiliated Hospital of Wenzhou Medical University. The exclusion criteria were as follows: (i) receipt of bridging therapy; (ii) acute myocardial infarction; (iii) severe liver or kidney damage; (iv) infection; (v) diagnosis of malignant tumour; (vi) rheumatic immune diseases; and (vii) incomplete baseline data or missing modified Rankin Scale (mRS) score at 3 months. Ultimately, 196 cases were included in this analysis.

Meanwhile, we incorporated 386 non-thrombolytic AIS patients between January 2020 and December 2020 from the Third Affiliated Hospital of Wenzhou Medical University. Patients diagnosed with AIS and possessing comprehensive medical records, along with follow-up information, were considered for inclusion. The exclusion criteria for this group were identical to those applied to patients receiving r-tPA treatment.

Individuals who underwent a health examination in October 2019 at the Third Affiliated Hospital of Wenzhou Medical University and met the following criteria were included as healthy controls (HCs): (i) free from any serious diseases and (ii) had NAR data. A total of 133 individuals met these criteria and participated in the study.

The study was approved by the Institutional Ethics Committee review board of the Third Affiliated Hospital of Wenzhou Medical University and was performed under the Declaration of Helsinki. All patients gave informed consent to participate in the study.

### Data collection

The admission demographics, including hypertension, diabetes, hyperlipidaemia and atrial fibrillation, were obtained from the electronic medical records. Blood routine examinations were performed using XT-1800i (Sysmex, Kobe, Japan). Blood biochemical examinations were performed using ARCHITECT c16000 (Abbott Laboratories, IL, USA). The blood routine and biochemical examinations were uniformly conducted early in the morning on the day following thrombolysis.

The recovery of neurofunction in stroke patients was measured using the mRS.^13^ The mRS is a 7-point scale that rates a patient’s level of disability or dependence based on their ability to perform daily activities. The scale ranges from 0 to 6, with higher scores indicating greater disability or dependence. The scale is based on the patient’s ability to carry out activities of daily living, such as self-care, mobility and communication. A score of 0 on the mRS indicates no symptoms, while a score of 6 indicates death. Scores between 1 and 5 represent varying degrees of disability or dependence, with 1 indicating no significant disability and 5 indicating severe disability requiring constant care. In our study, we employed two trained physicians to conduct phone interviews and collect the 3-month mRS data. We defined a score of 3 or higher as indicative of a poor prognosis, while a score of <3 was considered indicative of a good prognosis.^14,15^

The National Institutes of Health Stroke Scale (NIHSS) is a standardized assessment tool used to evaluate the severity of stroke symptoms in patients. It is widely used in clinical practice and research studies to measure the level of impairment caused by stroke.^16^ The NIHSS is a 15-item scale that assesses different areas of neurological function, including the levels of consciousness, language function, visual fields, motor function and sensation. Each item is scored based on the severity of the deficit, with scores ranging from 0 (no deficit) to 4 (severe deficit). The total score ranges from 0 to 42, with higher scores indicating more severe impairment. ΔNIHSS was calculated by subtracting the 24-hour NIHSS from the admission NIHSS, with positive values indicating clinical improvement.^17^ The neurologist assessed our patients’ NIHSS scores at hospital admission and 24 hours after admission.

The aetiology of AIS patients in our study was classified based on the Trial of ORG 10172 in Acute Stroke Treatment (TOAST) criteria, which includes large artery atherosclerosis (LAA), small-artery occlusion (SAO), cardioembolic (CE), stroke of other determined aetiology (SOE) and undetermined subtypes (SUE).^18^ Clinical tests such as CT, MRI, MRA, transcranial Doppler, carotid ultrasonography, electrocardiography and echocardiography were performed and analysed by neurologists to determine the TOAST classification. Once all required investigations were completed, patients were classified into one of the TOAST subtypes.

### Statistical analysis

NAR: Neutrophil count (×10^9^/L) to ApoA1 (g/L) ratio.

Statistical analysis was performed using SPSS Statistics 26.0 software (SPSS Inc., Chicago, IL). The percentage bar charts, scatter plots and box charts were generated using GraphPad Prism 9. The statistical tests were two-tailed, and the alpha-level that was used to determine the significance was 0.05. Continuous variables were examined for normality using the Kolmogorov–Smirnov test and reported as mean ± standard deviation or median and interquartile range, as appropriate. The *t*-test was employed for normally distributed and homogeneity of variance between groups, while the Mann–Whitney U-test was used for non-normally distributed cases. Based on quartiles of NAR, patients were divided into four groups (Q1: 1.243–2.937, Q2: 2.950–3.846, Q3: 3.864–5.068, Q4: 5.126–12.541) for further analysis. One-way ANOVA or Kruskal–Wallis H-test was employed for continuous variables, while chi-square or Fisher’s exact test was used for categorical variables with an expected frequency of 5 or <5 to compare the baseline characteristics across these groups. Non-parametric test and the paired-sample Wilcoxon signed-rank test were used to compare the admission NIHSS score and the 24-hour NIHSS score. Univariate logistic regression analysis was performed to identify potential confounders for the 3-month prognosis, and multivariate logistic regression analysis was used to adjust for variables with a *P*-value < 0.05 and TOAST in univariate analysis. The estimate odds ratios (ORs) with 95% confidence intervals (CIs) were calculated to explore the relationship between probable predictors and the 3-month prognosis. Model 1 represented the univariate analysis for NAR, model 2 adjusted for age and gender, and model 3 further added blood urea nitrogen, admission NIHSS and TOAST as covariates. Logistic regression analysis was also used in the outcome of haemorrhagic transformation (HT) and the 3-month prognosis of AIS patients treated without thrombolytic. Decision curve analysis was employed to further investigate the comparison of net benefits between NAR and neutrophil count in the practical application. The performance of NAR in predicting the three-month prognosis was evaluated using the receiver operating characteristic curve (ROC). The integrated discrimination improvement (IDI) and the net reclassification index (NRI) were calculated to evaluate the value of adding the neutrophil count or NAR after r-tPA treatment to the conventional model.

## Results

### Baseline characteristics

A total of 196 AIS patients with r-tPA and 133 HCs were included in the primary analysis. Table 1 presents the characteristics of the AIS patients with r-tPA and HCs. Compared to HCs, AIS patients with r-tPA were older (69.00 [57.25–77.75] versus 41.00 [35.00–47.00]; *P* < 0.001) and had a higher proportion of males (122 [62.24%] versus 68 [51.13%]; *P* = 0.045). Additionally, AIS patients with r-tPA had a significantly higher level of NAR (3.86 [2.94–5.11] versus 2.80 [2.16–3.81]; *P* < 0.001). The NAR in AIS patients treated with r-tPA remained higher than that in HCs even after age and gender matching (3.81 [2.91–4.56] versus 2.22 [1.83–2.76]; *P* < 0.001). To present more detailed information about the data, we created box plots with scatter points before and after matching (Supplementary Fig. 1). The outliers and their corresponding values for neutrophil count, ApoA1 levels and NAR before and after matching have been indicated.

**Table 1:** Demographic and laboratory characteristics of AIS patients with r-tPA and HCs

Variable	HCs	AIS	*P*-value
Before matching	*n* = 133	*n* = 196	
Age (years)	41.00 (35.00–47.00)	69.00 (57.25–77.75)	<0.001
Gender (male, n.%)	68 (51.13)	122 (62.24)	0.045
Neutrophil (×10^9^/L)	3.33 (2.58–4.31)	5.00 (3.90–6.30)	<0.001
ApoA1 (g/L)	1.17 (1.06–1.29)	1.32 (1.18–1.45)	<0.001
NAR	2.80 (2.16–3.81)	3.86 (2.94–5.11)	<0.001
After matching	*n* = 40	*n* = 40	
Age (years)	50.50 (46.00–56.00)	51.00 (46.25–56.75)	0.703
Gender (male, n.%)	26 (65.00)	26 (65.00)	1.000
Neutrophil (×10^9^/L)	2.98 (2.34–3.57)	4.85 (3.93–5.80)	<0.001
ApoA1 (g/L)	1.24 (1.14–1.47)	1.36 (1.19–1.45)	0.547
NAR	2.22 (1.83–2.76)	3.81 (2.91–4.56)	<0.001

AIS, acute ischaemic stroke; r-tPA, recombinant tissue plasminogen activator; HCs, healthy controls; ApoA1, apolipoprotein A1; NAR, neutrophil to apolipoprotein A1 ratio.

**Figure 2:**
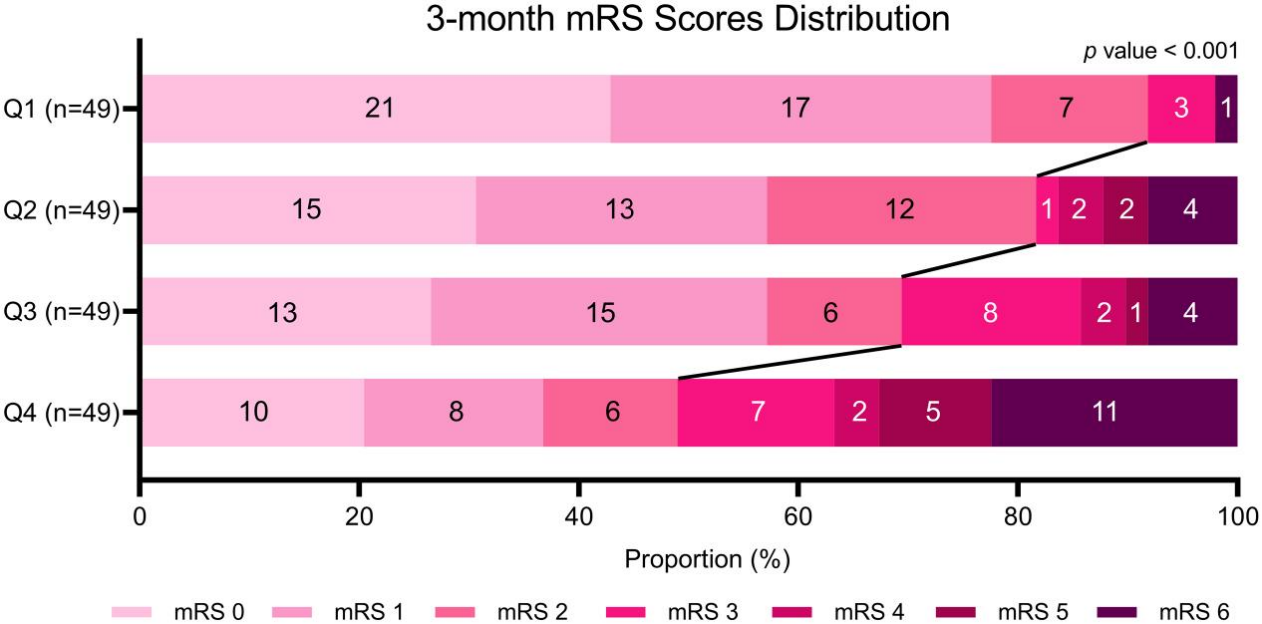
**mRS distribution at 3 months for the NAR quartiles.** A score of 3 or higher as indicative of poor prognosis, while a score of <3 was considered indicative of a good prognosis. The *P*-value was obtained from the chi-square test, F = 25.318, and there was a significant difference between good and poor prognosis from NAR quartiles (*P* < 0.001).

Subsequently, AIS patients with r-tPA were categorized into four groups based on quartiles of NAR for further analysis (Table 2). No significant differences were observed among these four groups with respect to age, gender, hypertension, diabetes, hyperlipidaemia, atrial fibrillation, blood urea nitrogen (BUN), uric acid (UA) and stroke subtypes. However, significant differences were observed in white blood cells (WBC), blood platelet count (PLT), creatinine, high density lipoprotein-cholesterol (HDL-C) and total cholesterol (TC) of the patients (*P* < 0.001, *P* = 0.005, *P* = 0.016, *P* < 0.001, *P* = 0.041, respectively). Pairwise comparisons using Bonferroni correction showed that there were significant differences in admission NIHSS between Q1 and Q4 (adjusted *P* < 0.001), as well as mRS scores between Q1 and Q4 (adjusted *P* < 0.001), while no significant differences were observed between the other groups. The relationship between NAR levels and prognosis was more evident in Fig. 2, where the proportion of AIS patients with high mRS scores increased as NAR levels increased (*P* < 0.001).

**Table 2:** Characteristics of AIS patients with r-tPA according to NAR quartiles

Variable	Total	NAR				*P*-value
		Q1 (1.243–2.937)	Q2 (2.950–3.846)	Q3 (3.864–5.068)	Q4 (5.126–12.541)	
Patients, *n*	196	49	49	49	49	
Age (years)	69.00 (57.25–77.75)	69.00 (58.50–76.00)	68.00 (55.50–76.50)	68.00 (56.00–77.50)	72.00 (59.50–82.00)	0.662
Gender (male, n.%)	122 (62.24)	29 (59.18)	24 (48.98)	34 (69.39)	35 (71.43)	0.083
Hypertension (n.%)	124 (63.27)	32 (65.31)	27 (55.1)	34 (69.39)	31 (63.27)	0.516
Diabetes (n.%)	39 (19.90)	7 (14.29)	10 (20.41)	9 (18.37)	13 (26.53)	0.493
Hyperlipidaemia (n.%)	10 (5.10)	3 (6.12)	3 (6.12)	4 (8.16)	0 (0.00)	0.232
Atrial fibrillation (n.%)	30 (15.31)	6 (12.24)	6 (12.24)	6 (12.24)	12 (24.49)	0.236
WBC (×10^9^/L)	7.40 (6.20–8.68)	5.60 (4.85–6.20)	6.80 (6.25–7.60)	7.90 (7.40–8.45)	9.60 (8.60–11.45)	<0.001
PLT (×10^9^/L)	195.06 ± 54.89	172.18 ± 51.89	196.45 ± 49.64	208.35 ± 47.18	203.24 ± 63.80	0.005
BUN (mmol/L)	4.80 (4.04–5.87)	4.76 (3.85–5.87)	4.70 (4.02–5.60)	4.74 (3.98–5.81)	5.23 (4.27–6.25)	0.309
Creatinine (μmol/L)	68.00 (62.00–76.00)	67.00 (62.00–72.00)	64.00 (58.00–76.00)	69.00 (63.00–79.00)	71.00 (68.00–80.50)	0.016
UA (μmol/L)	310.00 (260.00–383.00)	305.00 (242.50–364.50)	298.00 (256.25–379.25)	306.00 (272.00–388.00)	319.00 (268.00–403.00)	0.364
HDL-C (mmol/L)	1.05 (0.91–1.26)	1.19 (1.02–1.43)	1.07 (0.95–1.26)	1.00 (0.89–1.11)	0.95 (0.79–1.19)	<0.001
TC (mmol/L)	4.60 (3.98–5.26)	4.64 (3.89–5.23)	4.70 (4.09–5.57)	4.77 (4.18–5.38)	4.29 (3.41–4.76)	0.041
Admission NIHSS	7.00 (4.00–12.75)	5.00 (4.00–7.50)	7.00 (4.00–13.00)	7.00 (4.50–13.50)	10 (6.00–16.50)	<0.001
24-hour NIHSS	4.00 (2.00–8.00)	2.00 (1.00–4.00)	5.00 (3.00–8.50)	2.00 (5.00–9.50)	4.00 (7.00–14.00)	<0.001
ΔNIHSS	1.50 (0.00–4.00)	2.00 (1.00–4.00)	1.00 (0.00–4.50)	1.00 (0.00–4.00)	1.00 (0.00–4.00)	0.385
3-month mRS	1 (0–3)	1 (0–1)	1 (0–2)	1 (0–3)	3 (1–5)	<0.001
Stroke subtypes, *n* (%)						0.404
CE	65 (33.16)	13 (26.53)	18 (36.73)	14 (28.57)	20 (40.82)	
LAA	78 (39.80)	23 (46.94)	15 (30.61)	25 (51.02)	15 (30.61)	
SAO	37 (18.88)	10 (20.41)	10 (20.41)	6 (12.24)	11 (22.45)	
SOE/SUE	16 (8.16)	3 (6.12)	6 (12.24)	4 (8.16)	3 (6.12)	

AIS, acute ischaemic stroke; r-tPA, recombinant tissue plasminogen activator; NAR, neutrophil to apolipoprotein A1 ratio; WBC, white blood cells; PLT, blood platelet count; BUN, blood urea nitrogen; UA, uric acid; HDL-C, high density lipoprotein-cholesterol; TC, total cholesterol; NIHSS, National Institutes of Health Stroke Scale; ΔNIHSS, admission minus 24-hour NIHSS; mRS, modified Rankin Scale; CE, cardioembolism; LAA, large artery atherosclerosis; SAO, small-artery occlusion; SOE, stroke of other determined aetiology; SUE, stroke of undetermined aetiology.

We conducted individual comparisons between the HCs and the Q1–Q4 groups (Supplementary Table 1). The age of the Q1–Q4 groups was higher than that of the HCs group (all *P* < 0.05). The WBC count of the Q1 group was lower than that of the HCs group, while the Q2–Q4 groups had higher counts (all *P* < 0.05). Both PLT counts and HDL-C levels in the Q1–Q4 groups were lower than those in the HCs group (all *P* < 0.05). Significant differences in gender and creatinine levels were observed only in the Q3, Q4 to HCs group, which Q3, Q4 had higher proportions of males (*P* = 0.028, *P* = 0.014) and higher creatinine levels (*P* = 0.013, *P* = 0.001) than HCs. BUN and TC levels differed significantly only between the Q4 group and the HCs group, which Q4 had higher BUN levels (*P* = 0.021) and lower TC levels (*P* < 0.001) than HCs.

Furthermore, to indicate an improvement in patients’ conditions following r-tPA treatment for AIS, we employed a non-parametric test, the paired-sample Wilcoxon signed-rank test. This analysis revealed a significant decrease in the 24-hour NIHSS score (4.00, 2.00–8.00) compared to the admission NIHSS score (7.00, 4.00–12.25) (*P* < 0.001).

### NAR to predict the 3-month prognosis of AIS patients with r-tPA

The area under the curve of the ROC was 0.755 (95% CI 0.680–0.829, *P* < 0.001), indicating moderate predictive ability of NAR for 3-month prognosis in AIS patients. To further investigate the relationship between high NAR levels and poor prognosis, we conducted univariate regression analyses (Table 3) and found that the OR value of NAR was 1.720 (95% CI, 1.393–2.124; *P* < 0.001), which increased with 3-month mRS. Age (*P* < 0.001), gender (*P* = 0.022), WBC (*P* < 0.001), BUN (*P* = 0.002) and admission NIHSS (*P* < 0.001) were also observed to have strong associations with poor prognosis.

**Table 3:** Univariate logistic regression analyses of factors for 3-month poor outcome in AIS patients with r-tPA

Variables	Univariate logistic regression		
	OR	95% CI	*P*-value
Neutrophil	1.708	1.402–2.080	<0.001
ApoA1	0.894	0.216–3.698	0.877
NAR	1.720	1.393–2.124	<0.001
Age	1.076	1.043–1.110	<0.001
Gender	0.471	0.248–0.896	0.022
Hypertension	1.324	0.679–2.581	0.411
Diabetes	1.462	0.686–3.116	0.325
Hyperlipidaemia	1.166	0.290–4.684	0.829
Atrial fibrillation	2.033	0.903–4.574	0.087
WBC	1.528	1.282–1.821	<0.001
PLT	0.998	0.993–1.004	0.595
BUN	1.458	1.154–1.842	0.002
Creatinine	1.019	0.997–1.042	0.089
UA	0.997	0.993–1.001	0.104
HDL-C	0.963	0.288–3.216	0.951
TC	0.895	0.665–1.204	0.464
Admission NIHSS	1.241	1.159–1.330	<0.001
24-hour NIHSS	1.392	1.259–1.538	<0.001
△NIHSS	0.991	0.928–1.059	0.797
CE	1.650	0.859–3.171	0.133
LAA	1.103	0.581–2.094	0.765
SAO	0.573	0.235–1.398	0.221
SOE/SUE	0.361	0.079–1.647	0.188

AIS, acute ischaemic stroke; r-tPA, recombinant tissue plasminogen activator; OR, odds ratio; CI, confidence interval; WBC, white blood cells; PLT, blood platelet count; BUN, blood urea nitrogen; UA, uric acid; HDL-C, high density lipoprotein-cholesterol; TC, total cholesterol; ApoA1, apolipoprotein A1; NAR, neutrophil to apolipoprotein A1 ratio; NIHSS, National Institutes of Health Stroke Scale; ΔNIHSS, admission minus 24-hour NIHSS; CE, cardioembolism; LAA, large artery atherosclerosis; SAO, small-artery occlusion; SOE, stroke of other determined aetiology; SUE, stroke of undetermined aetiology.

To gain a deeper understanding of the connection between NAR levels and prognosis, we performed multivariate logistic regression analysis and adjusted for covariates with a *P*-value < 0.05 and stroke subtypes in the univariate analysis (Table 4). As neutrophils are the main leucocyte population counts accounting for 50–70% of WBC, we excluded WBC from the prognostic model. In model 1, the OR of patients in Q4 compared to Q1 was 11.719 (95% CI: 3.652–37.605, *P* < 0.001). After adjusting for age and gender (model 2), the OR of patients in Q4 was 17.438 (95% CI: 4.842–62.799, *P* < 0.001). After further adjusting for age, gender, admission NIHSS, BUN and stroke subtypes (model 3), the OR of NAR and 3-month prognostic outcomes in Q4 was 13.314 (95% CI: 2.878–61.596, *P* = 0.001). These results suggest that patients with higher NAR levels are more likely to develop poor prognostic outcomes.

**Table 4:** Adjusted odds ratio (95% confidence interval) for 3-month prognosis

	Variables	OR (95% CI)	*P*-value
Model 1	Q1	Ref.	
	Q2	2.531 (0.723–8.856)	0.146
	Q3	4.963 (1.511–16.306)	0.008
	Q4	11.719 (3.652–37.605)	<0.001
Model 2	Q1	Ref.	
	Q2	2.479 (0.657–9.362)	0.180
	Q3	7.116 (1.972–25.677)	0.003
	Q4	17.438 (4.842–62.799)	<0.001
Model 3	Q1	Ref.	
	Q2	2.346 (0.480–11.461)	0.292
	Q3	4.707 (1.063–20.847）	0.041
	Q4	13.314 (2.878–61.596)	0.001

Model 1 is univariate analysis. Model 2 is adjusted for age and gender. Model 3 is adjusted for age, gender, blood urea nitrogen, admission NIHSS and stroke subtypes.

Decision curve analysis was utilized to further explore the comparison of net benefits between NAR and neutrophil count in practical applications (Supplementary Fig. 2). Building upon the multivariate logistic regression model 3, the baseline model was adjusted for variables including age, gender, BUN, admission NIHSS and stroke subtypes. The NAR model and neutrophil model incorporated NAR and neutrophil count into the baseline model, respectively. As the threshold probability increased, the net benefit of an intervention based on the model’s results decreased. Between the NAR model and the neutrophil model, the net benefit of intervention guided by the NAR model’s results exhibited strong performance in most instances (particularly when the threshold probability ranged from 0.14 to 0.30), surpassing the performance of the neutrophil model.

Furthermore, the new model, which involved adding NAR after r-tPA treatment to the conventional model (including age, gender, admission NIHSS scores, hypertension, hyperlipidaemia and atrial fibrillation), was found a statistically significant improvement in discriminatory power and risk reclassification for 3-month poor outcomes in AIS patients treated with r-tPA. This improvement was evident through the categorical NRI (*P* = 0.035) of 12.9% and IDI (*P* = 0.013) of 5.2%. Meanwhile, the inclusion of neutrophil count into the conventional model resulted in a categorical NRI (*P* = 0.302) of 7.3% and an IDI (*P* = 0.008) of 6.3% (Table 5).

**Table 5:** Reclassification statistics (95% CI) for 3-month poor outcome in AIS patients with r-tPA

Model	Categorical NRI		IDI	
	Estimate (95% CI)	*P*-value	Estimate (95% CI)	*P*-value
Conventional model		Ref.		Ref.
Conventional model + neutrophil	0.073 (−0.066–0.211)	0.302	0.063 (0.016–0.110)	0.008
Conventional model + NAR	0.129 (0.009–0.250)	0.035	0.052 (0.011–0.092)	0.013

The conventional model included: age, gender, admission NIHSS scores, hypertension, hyperlipidaemia and atrial fibrillation. NRI, net reclassification index; IDI, integrated discrimination improvement; NAR, neutrophil to apolipoprotein A1 ratio.

To gain further insight, we performed subgroup analyses to explore whether the predictive value of NAR differs in different groups (Table 6). We divided AIS patients into subgroups according to median age, gender, admission NIHSS, hypertension, diabetes, atrial fibrillation and stroke subtypes. The results showed that the interaction between NAR and CE (*P* interaction = 0.032) or LAA (*P* interaction = 0.009) was significant. In univariate regression analyses, NAR had a strong association with poor prognosis in every subgroup (*P* < 0.05) except for LAA (*P* = 0.086).

**Table 6:** Subgroup analyses for the 3-month mRS by NAR

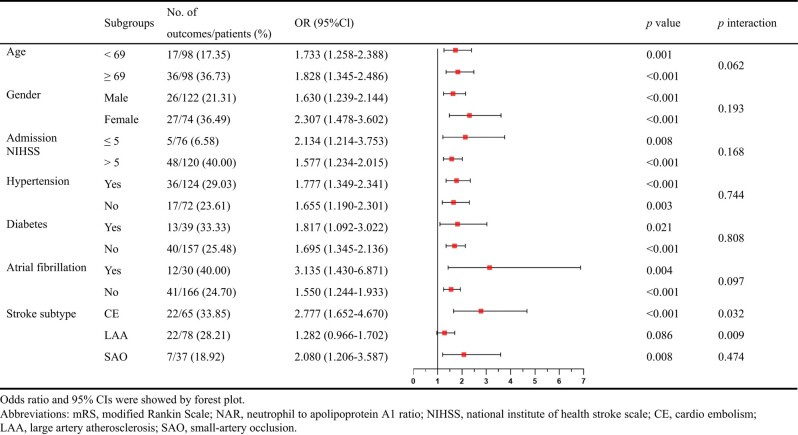

### Influencing factors of HT in AIS patients receiving r-tPA

We subdivided the primary study population, which consisted of AIS patients with r-tPA treatment, into two groups based on the occurrence of HT (Supplementary Table 2). The findings indicated that neutrophil count (*P* = 0.794), ApoA1 (*P* = 0.722) and NAR (*P* = 0.777) levels exhibited no significant differences between cases with and without HT. The group with HT has less in males (*P* = 0.015) and lower creatinine levels (*P* < 0.001) than the non-HT group. In the context of univariate logistic regression analysis using HT as the outcome (Supplementary Table 3), gender (95% CI 0.103–0.826, *P* = 0.020) and creatinine (95% CI 0.879–0.987, *P* = 0.017) emerged as the significant risk factors. Our findings indicated that neutrophil count was not a risk factor for HT (95% CI 0.772–1.252, *P* = 0.892).

### Prognostic factors and comparisons in AIS patients treated with r-tPA and non-thrombolytic

We classified AIS patients treated with r-tPA and non-thrombolytic into groups based on their 3-month prognosis (Supplementary Table 4). Irrespective of thrombolytic treatment, patients with unfavourable outcomes exhibited higher levels of neutrophil count, age, WBC count and admission NIHSS (all *P* < 0.001). We found that NAR was significantly different in the 3-month prognostic outcomes in both thrombolytic (4.72 [3.85–6.77] versus 3.60 [2.74–4.46], *P* < 0.001) and non-thrombolytic patients (3.39 [2.43–4.62] versus 2.73 [2.16–3.72], *P* < 0.001), growing high in worse outcomes, which mean an elevated NAR-value is inherent to any AIS with a poor prognosis.

Within the non-thrombolytic cohort, a univariate logistic regression was conducted using mRS 3–6 outcome (Supplementary Table 5). The outcomes demonstrated that neutrophil count, NAR, age, WBC count and admission NIHSS (all *P* < 0.05) were strongly associated with poor prognosis in non-thrombolytic patients.

Besides, we conducted a comparison between r-tPA patients and non-thrombolytic patients in terms of neutrophil count, ApoA1 and NAR levels in Supplementary Fig. 3. In comparison to non-thrombolytic patients, r-tPA patients exhibited higher neutrophil count (5.00 [3.90–6.30] versus 3.90 [3.20–5.30]; *P* < 0.001), lower ApoA1 (1.32 [1.18–1.45] versus 1.37 [1.20–1.56]; *P* = 0.001) and higher NAR levels (3.85 [2.94–5.11] versus 2.96 [2.20–4.03]; *P* < 0.001).

## Discussion

In this study, a higher NAR was shown to be associated with poor functional outcomes, even after adjusting for covariates and stroke subtypes, and we have identified the accuracy of NAR in predicting prognosis based on our data. To our knowledge, this is the first study that examines the relationship between a newly developed inflammatory marker, NAR, and the functional outcome of AIS.

Knowledge of predict factors relating to outcomes among the different TOAST subtypes will be useful to the selection of clinical management and thus help to improve prognosis. An analysis revealed significant differences in 90-day mortality rates among the different TOAST subtypes.^19^ In our study, according to TOAST classifications, 65 (33.16%) patients had a cardioembolic stroke, 78 (39.80%) patients had a large artery atherosclerosis stroke, 37 (18.88%) patients had a small-artery occlusion stroke, and 16 (8.16%) patients had a stroke of other determined aetiology or a stroke of undetermined aetiology. Our subgroup analysis showed that the NAR could be used as a predictor of CE and SAO but could not predict LAA. As the most common type of stroke, LAA is mainly caused by arterial stenosis or occlusion secondary to atherosclerosis, and most of these patients are complicated with basic diseases such as hypertension and diabetes.^20,21^ With the structure and function of blood vessels changing, vascular endothelial cell dysfunction plays a crucial role in the incidence and development of stroke.^22^ Some prospective cohort studies have suggested that HDL-C is negatively associated with ischaemic stroke, particularly with atherosclerotic stroke.^23,24^ But in the Asymptomatic Polyvascular Abnormalities Community study, little correlation between HDL-C levels and asymptomatic intracranial atherosclerotic stenosis (ICAS) was found.^25^ One possible hypothesis is that HDL-C levels may predict symptomatic ICAS but not asymptomatic ICAS. The progression of symptomatic ICAS differs from that of asymptomatic ICAS and that their predictors differ accordingly.^26^ As the major apolipoprotein in HDL-C, the predictive value of ApoA1 may be affected. Besides, different categories of LAA may account for the different predictive abilities of NAR for prognosis, probably due to differences in study population, sample size, imaging modalities and compounding effects of potential risk factors.

Consisting of serum neutrophil count and ApoA1, NAR is a composite inflammation and lipid metabolism marker. Neutrophils play an important role in innate immunity, which represent the largest proportion of the peripheral polymorphonuclear granulocytes (PMN).^27^ Within the first few hours after stroke onset, neutrophils are recruited to ischaemic sites, release neutrophil extracellular traps (NET) and other mediators that exacerbate tissue damage and poor neurologic improvement, potentially leading to adverse clinical outcomes.^7,28^ Besides, neutrophils are the primary source of matrix metalloprotein 9 (MMP-9), MMP-9 can directly damage the components of the blood-brain barrier and increase the risk of HT.^29^ ApoA1, a significant protein in the HDL complex, plays a role in regulating both the development and function of neutrophils. It downregulates neutrophil functions and reduces their activation, migration and adhesion, indicating a protective effect against inflammation.^30^ Neutrophils are involved in the acute inflammatory response, whereas ApoA1 exerts an anti-inflammatory effect, diminution of neutrophil degranulation and superoxide production.^31^ A single low dose infusion of ApoA1 administered after the onset of acute inflammation decreases neutrophil infiltration and inhibits neutrophil and endothelial cell activation.^32^ Therefore, the underlying mechanism of the association between elevated NAR and less favourable outcomes may involve the balance between overactive inflammation and protective regulation.

Our statistical results differ from existing literature that suggests a connection between neutrophil count and the risk of HT.^29^ Potential reasons for this discrepancy are as follows: (i) their recruited population included patients with pre-existing mRS < 2 and NIHSS > 5 points prior to the onset of disease, while we did not impose the same restrictions on our inclusion criteria. (ii) They focused on symptomatic intracranial haemorrhage (sICH), whereas our analysis pertained to HT, which encompassed sICH. Further evidence from multicentre studies with larger sample sizes and improved study designs is necessary to confirm the potential relationship between neutrophil count and the risk of HT.

We indicated a noteworthy elevation in NAR levels among individuals with unfavourable outcomes, irrespective of whether they were treated with thrombolytic or not. This underscores the value of NAR as a significant indicator for predicting poor prognosis in all cases of AIS. Nonetheless, it’s important to note that NAR levels were markedly higher in patients who received r-tPA compared to those who did not. This disparity may primarily be attributed to the fact that r-tPA induces neutrophil chemotaxis via the LRP-1/Akt pathway.^33^ The subsequent activation of neutrophils driven by r-tPA results in the observed elevation in NAR levels.

We conducted an in-depth exploration of the individual variability in r-tPA treatment, with recanalization outcomes indirectly inferred from the NIHSS score. The NIHSS has been shown to be a strong predictor of functional outcomes after endovascular recanalization.^34,35^ We observed an improvement in patients’ NIHSS scores 24 hours after thrombolysis compared to their scores upon admission. Additionally, we found no distinction in ΔNIHSS across NAR quartiles (*P* = 0.385) and demonstrated significant differences in ΔNIHSS for the three-month prognosis (*P* < 0.001). Future research could further explore the relationship between NAR and individualized r-tPA treatment outcomes. This may necessitate the consideration of additional clinical parameters, such as thrombus characteristics and neuroimaging manifestations, to better understand the role of NAR among different individuals’ responses to r-tPA treatment.

We have taken into account the technical and clinical viability of NAR measurements in the context of r-tPA and AIS. From a technical standpoint, the measurement of neutrophil counts and ApoA1 levels is routinely conducted in clinical laboratories using established techniques. In our study, NAR was calculated using the NAR. Neutrophil count was performed within 24 hours after admission using XT-1800i (Sysmex, Kobe, Japan), and ApoA1 was measured using ARCHITECT c16000 (Abbott Laboratories, IL, USA). NAR measurement is built upon routine blood tests, which are essential for hospitalized patients and can be conveniently calculated in real-time, thus demonstrating its clinical viability. Furthermore, the results produced by the sampling machine are accurate and widely used in major hospitals, thus highlighting the broad applicability of our findings.

There are several limitations of this study that should be acknowledged. Firstly, part of the data was collected retrospectively, and our study was conducted at a single centre. Secondly, the sample size was small, which limited the generalizability of our findings. In addition, we only collected NAR data after intravenous thrombolysis, and it would be beneficial to collect data at multiple time points. In our study, the calculation of NAR involved consistently collecting data from samples taken on the morning of the second day after thrombolysis. Nevertheless, neutrophils can exhibit substantial fluctuations following injury, shock and surgery. The variations in time between thrombolysis and NAR collection could potentially introduce differences in results. Subsequent research should delve deeper into this aspect for a comprehensive analysis. Further validation of the clinical applicability of NAR in larger clinical prospective cohorts is still needed. While our study suggested that NAR could serve as a potential biomarker for prognosis in AIS patients, the practical implementation of NAR-based stratification in the context of r-tPA therapy requires further investigation. Factors like the availability of rapid and reliable assays for neutrophil and apolipoprotein AI quantification, the standardization of measurement procedures and the potential impact of any pre-analytical variables need to be assessed. Future research efforts should aim to address the technical challenges and assess the clinical utility of NAR measurements, considering the dynamics of acute stroke treatment and the overall benefit they can offer to personalize patient care.

## Conclusions

Our research has demonstrated that a higher NAR level is significantly associated with a poor 3-month prognosis in AIS patients treated with r-tPA intravenous thrombolysis. Early monitoring of NAR levels may aid in risk stratification and treatment decision-making.

## Supplementary material

Supplementary material is available at *Brain Communications* online.

## Supplementary Material

fcae091_Supplementary_Data

## Data Availability

The data that support the findings of this study are available from the corresponding author upon reasonable request.
