# Supplementary material for: Predictive value of neutrophil to apolipoprotein A1 ratio in patients with acute ischaemic stroke
Source: Brain Commun. 2024 Mar 21;6(2):fcae091. doi: 10.1093/braincomms/fcae091 (PMC10977954; doi:10.1093/braincomms/fcae091)
Supplement: fcae091_Supplementary_Data [file fcae091_supplementary_data.docx]

Supplementary Table 1. Characteristics of AIS patients with r-tPA and HCs according to NAR quartiles.

| Variable |  | NAR | | | | *p* 1 | *p* 2 | *p* 3 | *p* 4 |
| --- | --- | --- | --- | --- | --- | --- | --- | --- | --- |
|  | HCs | Q1 (1.243-2.937) | Q2 (2.950-3.846) | Q3 (3.864-5.068) | Q4 (5.126-12.541) |  |  |  |  |
| Patients, n | 133 | 49 | 49 | 49 | 49 |  |  |  |  |
| Age (years) | 41.00 (35.00-47.00) | 69.00 (58.50-76.00) | 68.00 (55.50-76.50) | 68.00 (56.00-77.50) | 72.00 (59.50-82.00) | < 0.001 | < 0.001 | < 0.001 | < 0.001 |
| Gender (male, n.%) | 68 (51.13) | 29 (59.18) | 24 (48.98) | 34 (69.39) | 35 (71.43) | 0.334 | 0.797 | 0.028 | 0.014 |
| WBC (×10^9^/L) | 5.84 （4.94-7.03） | 5.60 (4.85-6.20) | 6.80 (6.25-7.60) | 7.90 (7.40-8.45) | 9.60 (8.60-11.45) | 0.035 | < 0.001 | < 0.001 | < 0.001 |
| PLT (×10^9^/L) | 233.00 (198.50-270.00) | 166.00 (136.50-213.50) | 186.00 (161.50-225.00) | 207.00 (176.50-235.00) | 208.00 (152.50-249.50) | < 0.001 | < 0.001 | 0.001 | 0.001 |
| BUN (mmol/L) | 4.63 (3.97-5.73) | 4.76 (3.85-5.87) | 4.70 (4.02-5.60) | 4.74 (3.98-5.81) | 5.23 (4.27-6.25) | 0.658 | 0.857 | 0.648 | 0.021 |
| Creatinine (μmol/L) | 64.00 (54.00-78.00) | 67.00 (62.00-72.00) | 64.00 (58.00-76.00) | 69.00 (63.00-79.00) | 71.00 (68.00-80.50) | 0.281 | 0.374 | 0.013 | 0.001 |
| UA (μmol/L) | 332.50 (256.00-400.50) | 305.00 (242.50-364.50) | 298.00 (256.25-379.25) | 306.00 (272.00-388.00) | 319.00 (268.00-403.00) | 0.127 | 0.366 | 0.914 | 0.810 |
| HDL-C (mmol/L) | 1.32 (1.15-1.54) | 1.19 (1.02-1.43) | 1.07 (0.95-1.26) | 1.00 (0.89-1.11) | 0.95 (0.79-1.19) | 0.016 | < 0.001 | < 0.001 | < 0.001 |
| TC (mmol/L) | 4.84 (4.28-5.65) | 4.64 (3.89-5.23) | 4.70 (4.09-5.57) | 4.77 (4.18-5.38) | 4.29 (3.41-4.76) | 0.076 | 0.450 | 0.454 | < 0.001 |

Abbreviations: AIS, acute ischemic stroke; r-tPA, recombinant tissue plasminogen activator; HCs, healthy controls; NAR, neutrophils to apolipoprotein A1 ratio; WBC, white blood cells; PLT, blood platelet count; BUN, blood urea nitrogen; UA, uric acid; HDL-C, high density lipoprotein-cholesterol; TC, total cholesterol.

Supplementary Table 2. Characteristics of AIS patients with r-tPA according to the occurrence of HT.

| Variable | Total | non-HT | HT | *p* value |
| --- | --- | --- | --- | --- |
| Patients, n | 195 | 178 | 17 |  |
| Neutrophil (×10^9^ /L) | 5.00 (3.90-6.30) | 5.00 (3.90-6.40) | 5.10 (3.75-6.05) | 0.794 |
| ApoA1 (g/L) | 1.32 ± 0.22 | 1.31 ± 0.23 | 1.33 ± 0.21 | 0.722 |
| NAR | 3.85 (2.94-5.11) | 3.88 (2.95-5.16) | 3.84 (2.77-5.17) | 0.777 |
| Age (years) | 69.00 (57.25-77.75) | 68.50 (57.00-77.00) | 76.00 (61.50-83.00) | 0.101 |
| Gender (male, n.%) | 122 (62.56) | 116 (65.17) | 6 (35.29) | 0.015 |
| Hypertension (n.%) | 124 (63.59) | 112 (62.92) | 12 (70.59) | 0.530 |
| Diabetes (n.%) | 38 (19.49) | 35 (19.66) | 3 (17.65) | 0.841 |
| Hyperlipidemia (n.%) | 10 (51.28) | 10 (5.62) | 0 (0.00) | 0.316 |
| Atrial fibrillation (n.%) | 30 (15.38) | 25 (14.04) | 5 (29.41) | 0.093 |
| WBC (×10^9^/L) | 7.40 (6.20-8.68) | 7.45 (6.20-8.63) | 6.80 (5.10-8.90) | 0.452 |
| PLT (×10^9^/L) | 195.06 ± 54.89 | 194.40 ± 54.62 | 206.18 ± 57.00 | 0.398 |
| BUN (mmol/L) | 4.80 (4.04-5.87) | 4.78 (4.07-5.80) | 5.59 (3.94-6.26) | 0.463 |
| Creatinine (μmol/L) | 68.00 (62.00-76.00) | 68.50 (62.75-77.25) | 64.0 (56.0-69.5) | < 0.001 |
| UA (μmol/L) | 310.00 (260.00-383.00) | 319.00 (260.00-386.00) | 286.00 (248.50-313.00) | 0.055 |
| HDL-C (mmol/L) | 1.05 (0.91-1.26) | 1.05 (0.91-1.25) | 1.06 (0.88-1.28) | 0.765 |
| TC (mmol/L) | 4.64 ± 1.08 | 4.68 ± 1.08 | 4.28 ± 1.06 | 0.147 |
| Admission NIHSS | 7.00 (4.00-12.75) | 7.00 (4.00-11.00) | 13.00 (4.50-17.00) | 0.190 |
| 24-hour NIHSS | 4.00 (2.00-8.00) | 4.00 (2.00-7.00) | 7.00 (2.50-13.00) | 0.167 |
| ΔNIHSS | 1.50 (0.00-4.00) | 2.00 (0.00-4.00) | 1.00 (0.00-5.50) | 0.944 |
| Stroke subtype, n (%) |  |  |  |  |
| CE | 64 (32.82) | 59 (33.15) | 5 (29.41) | 0.754 |
| LAA | 78 (40.00) | 70 (39.33) | 8 (47.06) | 0.534 |
| SAO | 37 (18.97) | 35 (19.66) | 2 (11.76) | 0.427 |
| SOE/SUE | 16 (8.21) | 14 (7.87) | 2 (11.76) | 0.576 |

Abbreviations: AIS, acute ischemic stroke; r-tPA, recombinant tissue plasminogen activator; ApoA1, apolipoprotein A1; NAR, neutrophil to apolipoprotein A1 ratio; WBC, white blood cells; PLT, blood platelet count; BUN, blood urea nitrogen; UA, uric acid; HDL-C, high density lipoprotein-cholesterol; TC, total cholesterol; NIHSS, national institute of health stroke scale; ΔNIHSS, admission minus 24-hour NIHSS; CE, cardio embolism; LAA, large artery atherosclerosis; SAO, small-artery occlusion; SOE, stroke of other determined etiology; SUE, stroke of undetermined etiology.

Supplementary Table 3. Univariate logistic regression analyses of factors for HT in AIS patients with r-tPA.

| Variables | Univariate logistic regression | | |
| --- | --- | --- | --- |
|  | OR | 95% CI | *p* value |
| Neutrophil | 0.983 | 0.772-1.252 | 0.892 |
| ApoA1 | 1.497 | 0.164-13.630 | 0.720 |
| NAR | 0.935 | 0.695-1.258 | 0.657 |
| Age | 1.030 | 0.988-1.074 | 0.162 |
| Gender | 0.292 | 0.103-0.826 | 0.020 |
| Hypertension | 1.414 | 0.477-4.193 | 0.532 |
| Diabetes | 0.876 | 0.238-3.214 | 0.841 |
| Atrial fibrillation | 2.550 | 0.827-7.860 | 0.103 |
| WBC | 0.902 | 0.702-1.159 | 0.420 |
| PLT | 1.004 | 0.995-1.013 | 0.397 |
| BUN | 1.011 | 0.813-1.258 | 0.918 |
| Creatinine | 0.932 | 0.879-0.987 | 0.017 |
| UA | 0.994 | 0.988-1.000 | 0.070 |
| HDL-C | 1.613 | 0.251-10.349 | 0.614 |
| TC | 0.694 | 0.423-1.138 | 0.148 |
| Admission NIHSS | 1.038 | 0.976-1.104 | 0.235 |
| 24-hour NIHSS | 1.046 | 0.979-1.117 | 0.185 |
| ΔNIHSS | 1.005 | 0.905-1.115 | 0.930 |
| CE | 0.840 | 0.283-2.497 | 0.754 |
| LAA | 1.371 | 0.505-3.723 | 0.535 |
| SAO | 0.545 | 0.119-2.493 | 0.434 |
| SOE/SUE | 1.562 | 0.324-7.531 | 0.579 |

Abbreviations: HT, hemorrhagic transformation; AIS, acute ischemic stroke; r-tPA, recombinant tissue plasminogen activator; OR, odds ratio; CI, confidence interval; ApoA1, apolipoprotein A1; NAR, neutrophil to apolipoprotein A1 ratio; WBC, white blood cells; PLT, blood platelet count; BUN, blood urea nitrogen; UA, uric acid; HDL-C, high density lipoprotein-cholesterol; TC, total cholesterol; NIHSS, national institute of health stroke scale; ΔNIHSS, admission minus 24-hour NIHSS; CE, cardio embolism; LAA, large artery atherosclerosis; SAO, small-artery occlusion; SOE, stroke of other determined etiology; SUE, stroke of undetermined etiology.

Supplementary Table 4. Characteristics of AIS patients with r-tPA and non-thrombolytic for 3-month outcome.

| Variable | r-tPA | | | non-thrombolytic | | |
| --- | --- | --- | --- | --- | --- | --- |
|  | mRS < 2 | mRS ≥ 3 | *p* value | mRS < 2 | mRS ≥ 3 | *p* value |
| Patients, n | 143 | 53 |  | 253 | 133 |  |
| Neutrophil (×10^9^ /L) | 4.60 (3.60-5.60) | 6.40 (5.00-8.30) | < 0.001 | 3.80 (3.20-4.80) | 4.50 (3.45-6.65) | < 0.001 |
| ApoA1 (g/L) | 1.32 ± 0.21 | 1.31 ± 0.25 | 0.878 | 1.37 (1.20-1.56) | 1.35 (1.20-1.57) | 0.835 |
| NAR | 3.60 (2.74-4.46) | 4.72 (3.85-6.77) | < 0.001 | 2.73 (2.16-3.72) | 3.39 (2.43-4.62) | < 0.001 |
| Age (years) | 66.00 (55.00-75.00) | 77.00 (67.00-83.00) | < 0.001 | 65.00 (57.00-73.00) | 72.00 (63.00-82.12) | < 0.001 |
| Gender (male, n.%) | 96 (67.13) | 26 (49.06) | 0.020 | 170 (67.19) | 77 (57.89) | 0.085 |
| Hypertension (n.%) | 88 (61.54) | 36 (67.92) | 0.410 | 209 (82.61) | 115 (86.47) | 0.327 |
| Diabetes (n.%) | 26 (18.18) | 13 (24.53) | 0.323 | 108 (42.69) | 64 (48.12) | 0.307 |
| Hyperlipidemia (n.%) | 7 (4.89) | 3 (5.66) | 0.829 | 73 (28.85) | 35 (26.32) | 0.598 |
| Atrial fibrillation (n.%) | 18 (12.59) | 12 (22.64) | 0.082 | 9 (3.56) | 10 (7.52) | 0.087 |
| WBC (×10^9^/L) | 6.90 (5.80-8.10) | 8.70 (7.40-10.40) | < 0.001 | 6.25 (5.30-7.5) | 7.00 (5.60-8.75) | < 0.001 |
| PLT (×10^9^/L) | 196.32 ± 55.52 | 191.64 ± 53.51 | 0.597 | 202.00 (176.00-246.50) | 208.00 (166.50-263.00) | 0.845 |
| BUN (mmol/L) | 4.60 (3.97-5.55) | 5.50 (4.28-6.30) | < 0.001 | 4.77 (3.86-5.59) | 4.42 (3.81-5.71) | 0.406 |
| Creatinine (μmol/L) | 68.00 (62.00-76.00) | 69.00 (63.50-78.00) | 0.343 | 67.00 (62.25-73.75) | 66.00 (59.00-74.00) | 0.315 |
| UA (μmol/L) | 320.00 (266.00-383.50) | 291.50 (246.25-344.50) | 0.069 | 322.00 (268.00-388.75) | 319.00 (262.00-397.00) | 0.679 |
| HDL-C (mmol/L) | 1.06 (0.93-1.25) | 1.03 (0.89-1.32) | 0.672 | 0.95 (0.84-1.17) | 0.98 (0.81-1.18) | 0.792 |
| TC (mmol/L) | 4.68 ± 1.08 | 4.55 ± 1.07 | 0.465 | 4.47 ± 1.02 | 4.51 ± 1.18 | 0.689 |
| Admission NIHSS | 6.00 (4.00-9.00) | 14.00 (8.00-19.50) | < 0.001 | 2.00 (1.00-4.00) | 6.00 (4.00-8.75) | < 0.001 |
| 24-hour NIHSS | 3.00 (1.00-5.00) | 12.00 (7.00-18.00) | < 0.001 | - | - | - |
| ΔNIHSS | 2.00 (0.00-4.00) | 0.00 (0.00-4.00) | < 0.001 | - | - | - |
| Stroke subtype, n (%) |  |  |  | - | - | - |
| CE | 43 (30.07) | 22 (41.51) | 0.031 | - | - | - |
| LAA | 56 (39.16) | 22 (41.51) | 0.765 | - | - | - |
| SAO | 30 (20.98) | 7 (13.21) | 0.217 | - | - | - |
| SOE/SUE | 14 (9.79) | 2 (3.77) | 0.172 | - | - | - |

Abbreviations: AIS, acute ischemic stroke; r-tPA, recombinant tissue plasminogen activator; ApoA1, apolipoprotein A1; NAR, neutrophil to apolipoprotein A1 ratio; WBC, white blood cells; PLT, blood platelet count; BUN, blood urea nitrogen; UA, uric acid; HDL-C, high density lipoprotein-cholesterol; TC, total cholesterol; NIHSS, national institute of health stroke scale; ΔNIHSS, admission minus 24-hour NIHSS; CE, cardio embolism; LAA, large artery atherosclerosis; SAO, small-artery occlusion; SOE, stroke of other determined etiology; SUE, stroke of undetermined etiology.

Supplementary Table 5. Univariate logistic regression analyses of factors for 3-month poor outcome in AIS patients without r-tPA.

| Variables | Univariate logistic regression | | |
| --- | --- | --- | --- |
|  | OR | 95% CI | *p* value |
| Neutrophil | 1.302 | 1.158-1.464 | < 0.001 |
| ApoA1 | 1.189 | 0.512-2.760 | 0.687 |
| NAR | 1.388 | 1.192-1.617 | < 0.001 |
| Age | 1.048 | 1.029-1.069 | < 0.001 |
| Gender | 0.684 | 0.443-1.005 | 0.086 |
| Hypertension | 1.345 | 0.743-2.435 | 0.328 |
| Diabetes | 1.245 | 0.817-1.898 | 0.308 |
| Hyperlipidemia | 0.881 | 0.549-1.412 | 0.598 |
| Atrial fibrillation | 2.204 | 0.873-5.566 | 0.094 |
| WBC | 1.189 | 1.074-1.317 | 0.001 |
| PLT | 1.000 | 0.996-1.003 | 0.802 |
| BUN | 1.053 | 0.945-1.174 | 0.349 |
| Creatinine | 1.005 | 0.994-1.015 | 0.376 |
| UA | 1.000 | 0.998-1.002 | 0.962 |
| HDL-C | 1.529 | 0.682-3.426 | 0.303 |
| TC | 1.041 | 0.857-1.264 | 0.688 |
| Admission NIHSS | 1.745 | 1.539-1.979 | < 0.001 |

Abbreviations: AIS, acute ischemic stroke; r-tPA, recombinant tissue plasminogen activator; ApoA1, apolipoprotein A1; NAR, neutrophil to apolipoprotein A1 ratio; WBC, white blood cells; PLT, blood platelet count; BUN, blood urea nitrogen; UA, uric acid; HDL-C, high density lipoprotein-cholesterol; TC, total cholesterol; NIHSS, national institute of health stroke scale.


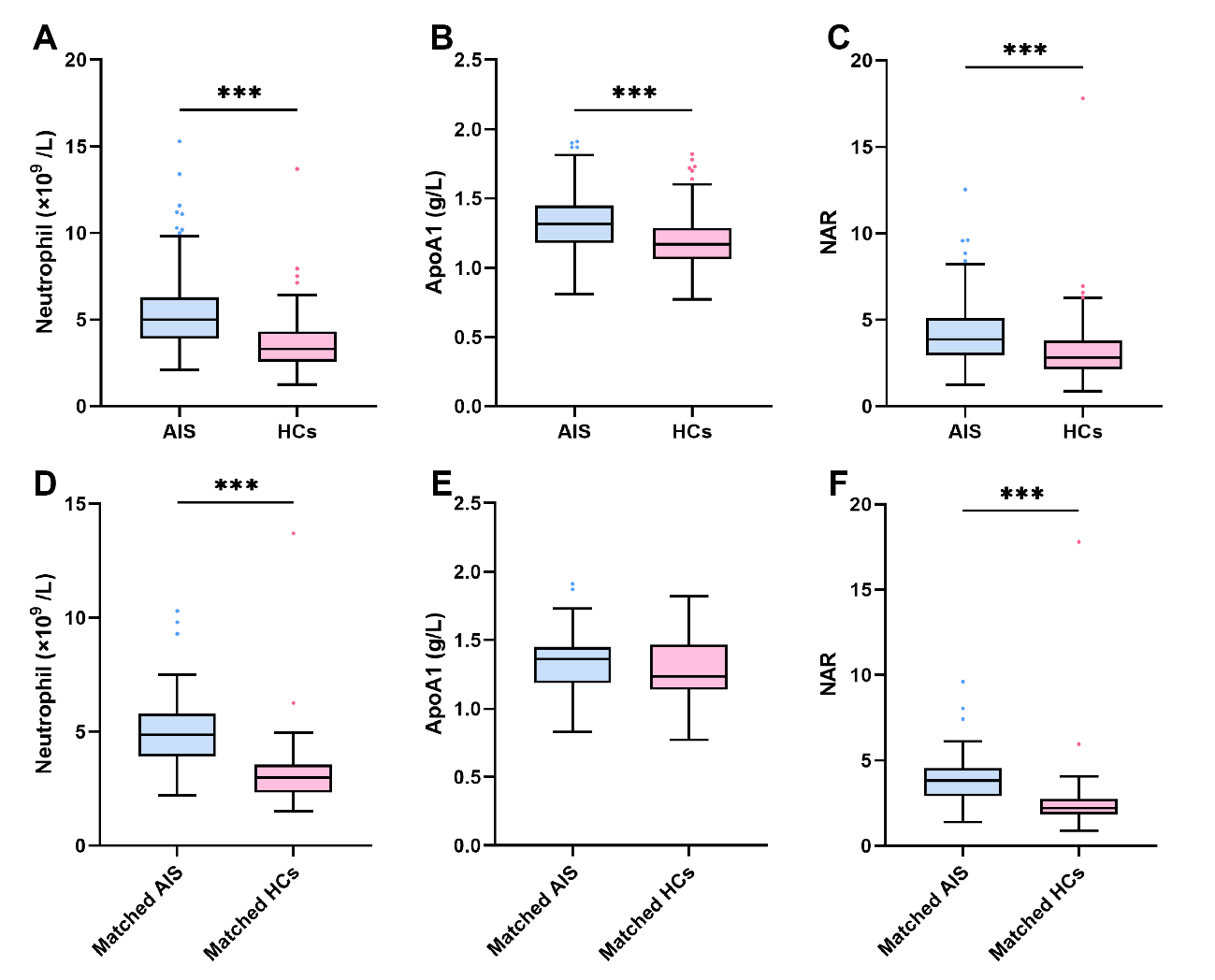


**Supplementary Figure 1: Box plots in r-tPA AIS patients and HCs before and after matching**

A: Neutrophil levels in r-tPA AIS patients and HCs. B: ApoA1 levels in r-tPA AIS patients and HCs. C: NAR levels in r-tPA AIS patients and HCs. D: Neutrophil levels in r-tPA AIS patients and HCs after age and sex matching. E: ApoA1 levels in r-tPA AIS patients and HCs after age and sex matching. F: NAR levels in r-tPA AIS patients and HCs after age and sex matching. The T-test was employed for normally distributed and homogeneity of variance between groups, while the Mann-Whitney U test was used for non-normally distributed cases. *p* value style: *p* ≤ 0.001 was represented by "***", *p* ≤ 0.01 was represented by "**", *p* ≤ 0.05 was represented by "*"


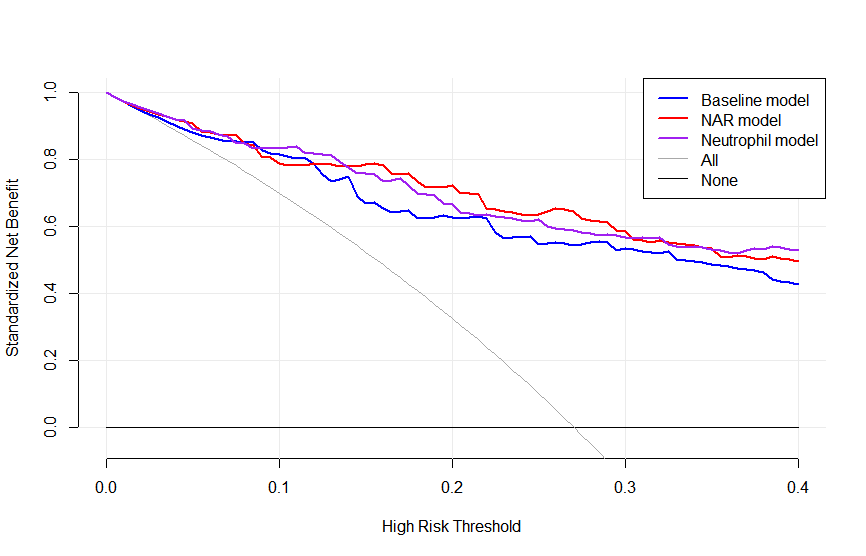


**Supplementary Figure 2: Decision curve analysis in the comparison of net benefits between NAR model and neutrophil model in practical application.**

Building upon the multivariate logistic regression model 3, the baseline model was adjusted for variables including age, gender, BUN, admission NIHSS, and stroke subtypes. The NAR model and neutrophil model incorporated NAR and neutrophils into the baseline model, respectively. As the threshold probability increased, the net benefit of an intervention based on the model's results decreased. Between the NAR model and the neutrophil model, the net benefit of intervention guided by the NAR model's results exhibited strong performance in most instances (particularly when the threshold probability ranged from 0.14 to 0.30), surpassing the performance of the neutrophil model.


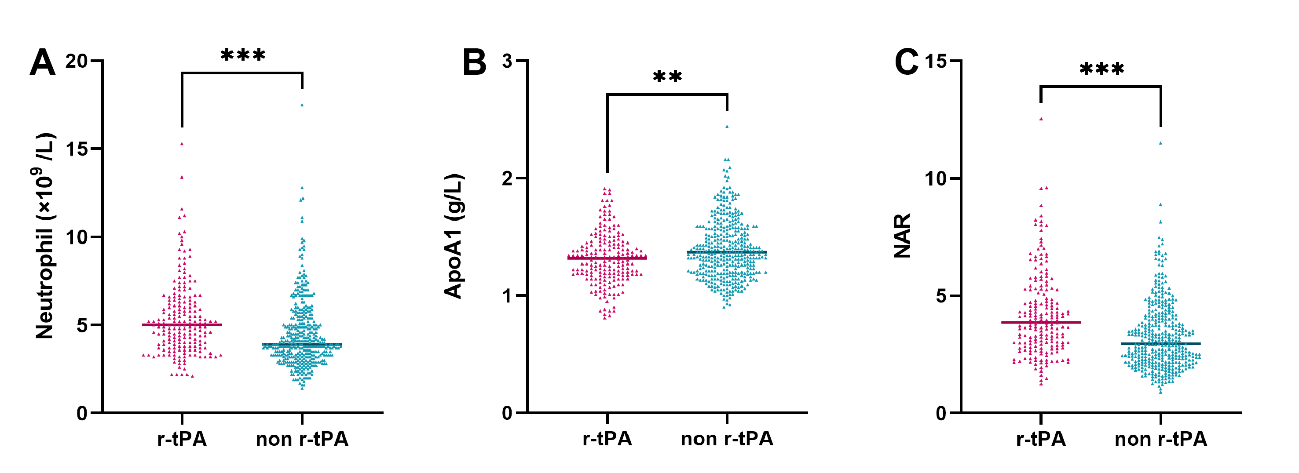


**Supplementary Figure 3: Scatter plots comparing indicators between r-tPA AIS patients and non-thrombolytic AIS patients.**

A: Neutrophil levels in r-tPA AIS patients and non-thrombolytic AIS patients. B: ApoA1 levels in r-tPA AIS patients and non-thrombolytic AIS patients. C: NAR levels in r-tPA AIS patients and non-thrombolytic AIS patients. Mann-Whitney U test was used to compare two groups’s indicators. p value style: *p* ≤ 0.001 was represented by "***", *p* ≤ 0.01 was represented by "**", *p* ≤ 0.05 was represented by "*"
